# Long Intergenic Noncoding RNA 00261 Acts as a Tumor Suppressor in Non-Small Cell Lung Cancer via Regulating miR-105/FHL1 Axis

**DOI:** 10.7150/jca.32251

**Published:** 2019-10-19

**Authors:** Zhiqiang Wang, Jiru Zhang, Bo Yang, Runsheng Li, Linfang Jin, Zhenjun Wang, Haifeng Yu, Chuanxin Liu, Yong Mao, Qingjun You

**Affiliations:** 1 Department of Thoracic and Cardiovascular Surgery, Affiliated Hospital of Jiangnan University, Wuxi, Jiangsu, 214062, China.; 2 Department of Anesthesiology, Affiliated Hospital of Jiangnan University, Wuxi, Jiangsu, 214062, China.; 3 Department of Radiotherapy, Affiliated Hospital of Jiangnan University, Wuxi, Jiangsu, 214062, China.; 4 Department of Respiratory Medicine, Affiliated Hospital of Jiangnan University, Wuxi, Jiangsu, 214062, China.; 5 Department of Pathology, Affiliated Hospital of Jiangnan University, Wuxi, Jiangsu, 214062, China.; 6 Department of Oncology, Affiliated Hospital of Jiangnan University, Wuxi, Jiangsu, 214062, China.

**Keywords:** Non-small cell lung cancer, LINC00261, miR-105, FHL1

## Abstract

**Purpose:** Long noncoding RNAs (lncRNAs) have recently received more attention for their roles in tumor progression. LINC00261 was studied in this research to identify how it affects the progression of non-small cell lung cancer (NSCLC).

**Methods:** Firstly, the expression of LINC00261 in NSCLC cells and paired samples of NSCLC tissue was detected by RT-qPCR. Then, the associations between LINC00261 expression level and clinicopathological characteristics were evaluated. Furthermore, functional assays of cell proliferation, colony formation and transwell, as well as western blot assay, luciferase assay and RNA immunoprecipitation (RIP) assay were conducted. Afterwards, the effects of LINC00261 expression on NSCLC formation and growing were confirmed by *in vivo* models.

**Results:** As results, expression of LINC00261 was significantly down-regulated in tumor samples than that in normal samples, which was correlated with the lymphatic metastasis, tumor size, tumor stage as well as patient survival time. Knockdown of LINC00261 inhibited tumor growth and invasion ability *in vitro*. In addition, miR-105 was identified as a direct target of LINC00261 via mechanism experiments and its expression in tumor tissues negatively correlated to LINC00261 expression. Further experiments found that Four and expression of Half LIM domains 1 (FHL1) was negatively correlated with miR-105 but positively with LINC00261. Moreover, *in vivo* assays verified the overexpression of LINC00261 could suppress formation of NSCLC and regulate the expression of miR-105/FHL1 axis.

**Conclusions:** These results indicate that LINC00261 could suppress metastasis and proliferation of NSCLC via suppressing miR-105/FHL1 axis, which may offer a new vision for interpreting the mechanism of NSCLC development.

## Introduction

As the third general cancer among human malignant tumors in 2015 1, the incidence rate of NSCLC remains high both in male and in female worldwide. In the last decades, although molecular targeted therapy is available for NSCLC patients, only a small part of patients' benefits from those discovered driver mutations 2. Therefore, further exploring the mechanisms of these genomic changes in NSCLC is urgently required.

LINC00261 has been shown to exert as a molecular biomarker in several cancers. For example, LINC00261 may be a useful biomarker of the prognosis for HCC patients, based on bioinformatics analysis 3. Down regulation of LINC00261 may contribute to the carcinogenesis and development of laryngeal carcinoma 4. However, it remains unknown the roles of LINC00261 in NSCLC.

Recently, plentiful studies have revealed the interaction between lncRNAs and miRNAs in several kinds of tumors which probably was associated with many oncogenes that had been known. LncRNA NEAT1 enhances the progression of colorectal cancer via competitively binding miR-34a with SIRT1 5. SP1-induced lncRNA-ZFAS1 is implicated in the progression of colorectal cancer via miR-150/VEGFA 6. LncRNA DGCR5 inhibits hepatocellular carcinoma development via regulating the miR-346/KLF14 axis 7. Shen *et al*. revealed that LncRNA MEG3 acts as a competing endogenous RNA to modulate HOXA11 via sponging miR-181a in myeloma 8. LINC00888 enhances tumorigenicity of melanoma through miR-126/CRK 9. However, the effect of this interaction on NSCLC progression is still unclear.

In this study, LINC00261 was found decreased in NSCLC samples. Besides, it inhibited invasion and proliferation *in vitro*. What's more, we further discovered the interaction between LINC00261 and miR-105 as well as the possible mechanism and confirmed the inhibition effect of LINC00261 on tumorgenesis of NSCLC *in vivo*.

## Materials and Methods

### Clinical samples and cell lines

60 NSCLC patients were enrolled for human tissues who received surgery at Affiliated Hospital of Jiangnan University. Before operation, written informed consent was achieved. The exclusion criteria were prior chemotherapy or radiotherapy. Tumor tissues and adjacent tissues were collected during the surgery and then immediately stored at -80°C. All tissues were analyzed by an experienced pathologist and the clinical data of those patients were recorded and analyzed (as shown in Table 1). The overall survival was calculated from the day of primary surgery to death or last follow-up. This study conforms to requirements of the Ethics Committee of Affiliated Hospital of Jiangnan University.

A549, SPCA1, H1975, H1299 and PC-9, and 16HBE (human normal bronchial epithelial cell) and 293T embryonic kidney cell line (American Type Culture Collection) were used in this study. Culture medium was consisted of penicillin, RPMI-1640 medium (Thermo Fisher Scientific, USA) and 10% fetal bovine serum (FBS; Invitrogen Life Technologies). Besides, cells were cultured at 37 ˚C in a humidified incubator containing 5% CO_2_.

### Cell transfection

After synthesized, lentiviral small hairpin RNA (shRNA) targeting LINC00261 was cloned into the pLenti-EF1a-EGFP-F2A-Puro vector (BiosettiaInc. San Diego, CA, USA). 293T cells were adopted for packaging LINC00261 lentivirus (LINC00261), the viruses and the empty vector (control). A549 cells were transfected by miRNA mimics and inhibitor provided by Genepharma Co., Ltd. (Shanghai, China). siRNA Universal Negative Control (Sigma-Aldrich) was used as controls.

### RNA extraction and qRT-PCR

Total RNA was extracted from macrodissected tissue samples or cells by using TRIzol reagent (Invitrogen, USA), and then was reverse-transcribed to cDNAs via reverse Transcription Kit (Takara Biotechnology Co., Ltd., Dalian, China). The β-actin and U6 served as the internal controls for LINC00261 and mi-RNA. Available primers used for SYBR Green real-time PCR were as follows: LINC00261, forwards 5'- ACATTTGGTAGCCCGTGGAG -3' and reverse 5'- TCTTCCCCGGAGAACTAGCA-3'; miR-105, forward 5′- GCCCTCG AGATACCATATCTATCCCCTTTTTCA-3′, and reverse 5′- GCCGAATTC CAACCATGAAGATACGAATTGATG-3′;β-actin, forward 5'-TTGTTACAGGAAGTCCCTTGCC-3' and reverse 5'-ATGCTATCACCTCCCCTGTGTG-3'. U6, forward 5'-CTCGCTTCGGCAGCACATATACT-3' and reverse 5'-ACGCTTCACGAATTTGCGTGTC-3'; Thermal cycle was as follows: 30 sec at 95˚C, 5 sec at 95˚C for 40 cycles, 35 sec at 60˚C.

### Western blot analysis

Reagent RIPA (Beyotime) was utilized to extract protein from cells. BCA protein assay kit (Takara) was chosen for quantifying protein concentrations. The target proteins were separated by SDS-PAGE. Then they were incubated with antibodies after replaced to the polyvinylidene fluoride (PVDF) membrane. Rabbit anti-β-actin and rabbit anti-FHL1, as well as goat anti-rabbit secondary antibody were purchased from Cell Signaling Technology (CST, USA). Chemiluminescent film was applied for assessment of protein expression with Image J software.

### Luciferase assays and RNA immunoprecipitation (RIP) assay

In our study, pGL3 vector (Promega) was used as a backbone for the construction of of 3ʹ-UTR of FHL1 or LINC00261, wild-type (WT) 3ʹ-UTR. Quick-change site-directed mutagenesis kit (Stratagene, Cedar Creek, USA) was adopted for site-directed mutagenesis of miR-105 binding site in FHL1 or LINC00261 3ʹ-UTR, mutant (MUT) 3ʹ-UTR. WT-3ʹ-UTR or MUT-3ʹ-UTR and miR-ctrl or miR-105 mimics was used for cell transfection. 48h later, the dual luciferase reporter assay system (Promega) was utilized to perform the luciferase assays. RIP assay was performed utilizing Magna RIP RNA-Binding Protein Immunoprecipitation Kit (Millipore). Co-precipitated RNAs were detected via RT-qPCR.

### Ethynyl deoxyuridine (Edu) Analysis

Proliferating cells were determined by using the 5-ethynyl-2'-deoxyuridine (EdU) abeling/detection kit (Ribobio, Guangzhou, China) according to the manufacturer's protocol. After 48 h of cultivation, the treated cells were incubated with 50 μM EdU labeling medium for 2 h at 37°C under 5% CO_2_ and fixed with 4% paraformaldehyde (pH 7.4) for 30 min, and then treated with 0.5% Triton X-100 for 20 min at room temperature. After washing with PBS, staining with anti-EdU working solution was performed at room temperature for 30 min. Cells were incubated with 100 μL Hoechst 33342 (5 μg/mL) at room temperature for 30 min, followed by observation under a fluorescent microscope. The percentage of EdU-positive cells was calculated from five random fields in three wells.

### Colony Formation assay

After cultured with FBS for 14 days in a six-well plate, all the cells were fixed with methanol and stained with 0.1% crystal violet. Meanwhile, numbers of colonies were counted for comparison.

### Wound healing assay

A549 and SPCA1 cell lines were collected for wound healing assay. Firstly, cells were transferred into 6-well plates, and then cultured in DMEM medium overnight. After scratched with a plastic tip, cells were cultured in serum-free DMEM. Wound closure was viewed at 24 h and 48 h later. Each assay was independently repeated in triplicate.

### Matrigel assay

5 ×10^4^ cells in 200µL serum-free RPMI-1640 were transformed to top chamber of an insert (8μm pore size; Millipore) which was coated with 50 µg Matrigel (BD Biosciences). And the bottom chamber was added RPMI-1640 and FBS. 48h later, after wiped by cotton swab, the top surface of chambers was immersed for 10 min with precooling methanol. Following were stain in crystal violet for 30 min. Three fields were used to count the data for invasion membrane.

### Xenograft model

The research was approved by the Animal Ethics Committee of Shandong University Animal Center. A549 cells (6×10^5^/mL) transfected with LINC00261 or control were implanted into both axillae of NOD/SCID mice (4-5 weeks old) subcutaneously. Tumor diameters were detected every 5 days. Tumor volume was calculated as the formula (Volume was calculated as length × width^2^ × 1/2). Mice were sacrificed and the grafts were removed after 4 weeks. Afterwards, the tumor tissues were got and prepared for Western Blot and qRT-PCR assay to detect the protein level of FHL1 and the RNA level of miR-105 in mice from different groups.

### Statistical analysis

SPSS 17.0 (SPSS, Chicago, IL, USA) was utilized to statistical analyze. Results were presented as mean ± standard error of the mean. Chi-square test, ANOVA test and Kaplan-Meier method were selected for clinical data analysis when appropriate. The data in figures were analyzed using a paired Student's t test. p values < 0.05 were considered significant.

## Results

### LINC00261 level in tissues and cells of NSCLC

**Figure 1:**
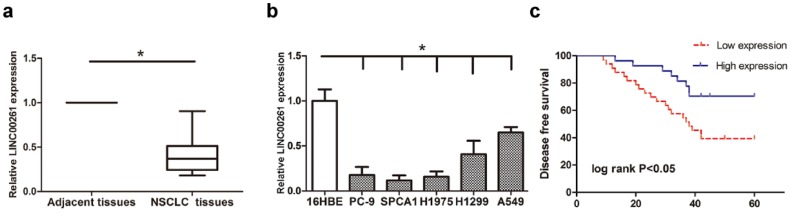
** Expression level of LINC00261 was decreased in NSCLC tissues and cell lines, and was associated with better disease-free survival of NSCLC patients** (a) LINC00261 expression was significantly decreased in the NSCLC tissues compared with adjacent tissues. (b) Expression levels of LINC00261 relative to GAPDH were determined in the human NSCLC cell lines and 16HBE (human bronchial epithelial cell) by RT-qPCR. (c) High level of LINC00261 was associated with better disease-free survival of patients with NSCLC. Data are presented as the mean ± standard error of the mean. *P<0.05.

Firstly, RT-qPCR was conducted for detecting LINC00261 expression in 60 patients' tumor tissues and 5 NSCLC cells. As the results, LINC00261 was significantly downregulated in tumor tissue samples compared to the adjacent tissues (Fig. 1a). Meanwhile, LINC00261 level was lower in NSCLC cells than that in normal human bronchial epithelial cell (Fig. 1b). Analysis of clinicopathological features in those patients demonstrated that downregulated LINC00261 obviously correlated to tumor size, lymph node metastasis and tumor stage (Table 1). Moreover, the patients were divided into two groups according to the median expression of LINC00261, and then the survival curves were estimated using the Kaplan-Meier analysis. The results showed that patients had a better disease-free survival with higher LINC00261 level (Fig. 1c).

### Overexpression of LINC00261 inhibited cell proliferation and invasion *in vitro*

**Figure 2:**
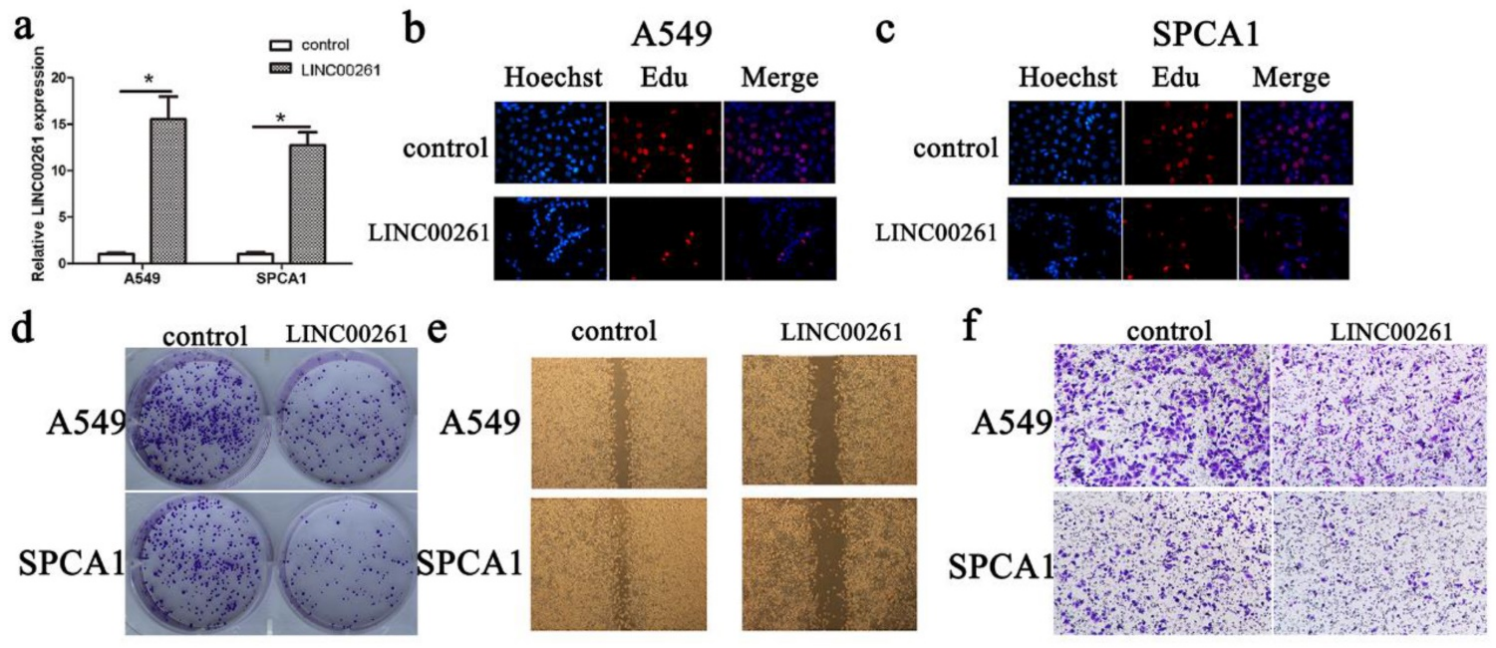
** Overexpression of LINC00261 decreased NSCLC cell proliferation migration and invasion** (a) LINC00261 expression in cancer cells transduced with empty vector (control) or LINC00261 virus (LINC00261) was detected by RT-qPCR. β-actin was used as an internal control. (b) Edu assay showed that overexpression of LINC00261 significantly decreased cell proliferation in A549 cancer cells. (c) Edu assay showed that knockdown of LINC00261 significantly decreased cell proliferation in SPCA1 cancer cells. (d) Colony formation assay demonstrated that oncogenic survival of cancer cells in LINC00261 group was significantly decreased compared with control group. (e) Wound healing assay showed that the migrated ability was obviously inhibited in LINC00261 group compared with control group. (f) Transwell assay showed that number of invaded cells in LINC00261 group was obviously reduced compared with control group. The results represent the average of three independent experiments (mean ± standard error of the mean). *P<0.05, as compared with the control cells.

According to LINC00261 expression in cancer cells, we chose A549 and SPCA1 cancer cells for overexpression of LINC00261. The LINC00261 lentiviruses (LINC00261) and the empty vector (control) were synthetized and transduced into these two cells. Then the LINC00261 expression was determined by qRT-PCR (Fig. 2a). Furthermore, the results of Edu assay showed that cell proliferation of NSCLC cells was inhibited after LINC00261 overexpressed (Fig. 2 b, c). Meanwhile, cancer formation assay showed that numbers of colonies were less in LINC00261 group than in control group (Fig. 2d). Then, we performed wound healing assay and transwell assay, and found that overexpressed LINC00261 suppressed NSCLC cell migration and invasion (Fig. 2 e, f).

### LINC00261 inhibited NSCLC tumorigenesis via miR-105/FHL1 axis

**Figure 4:**
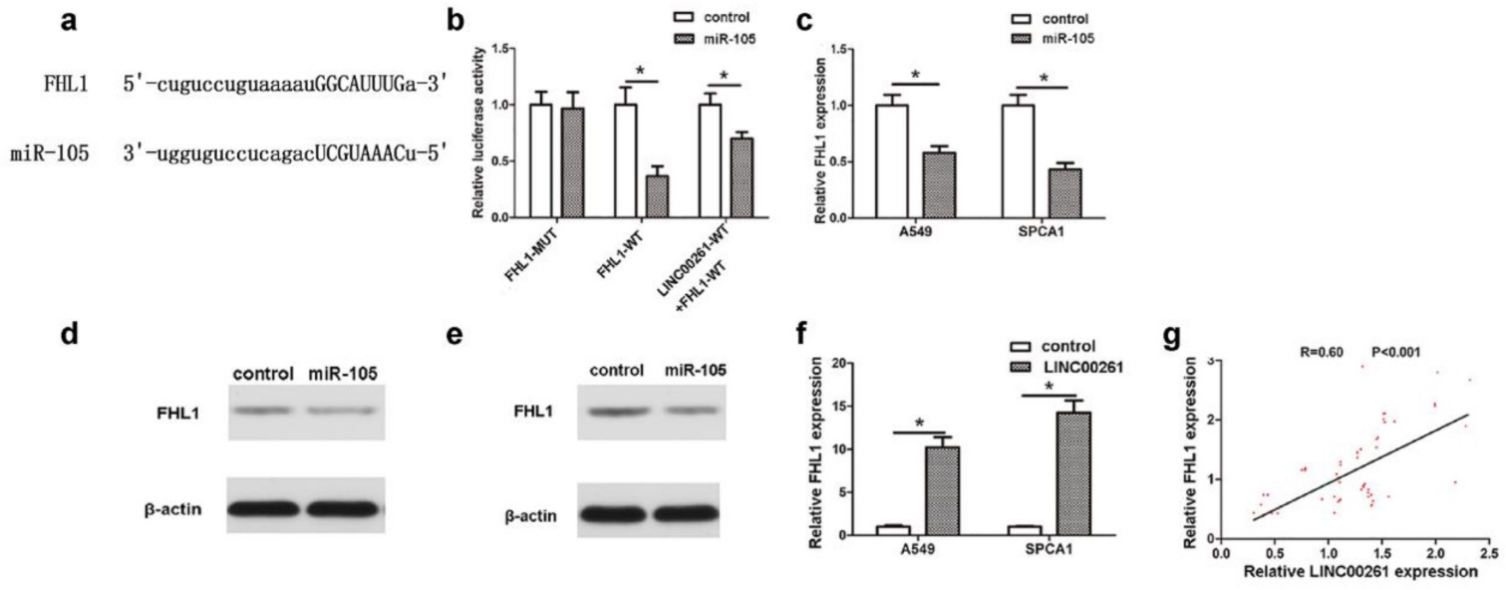
** MiR-105/FHL1 axis mediated the effect of LINC00261 on NSCLC cells** (a) By using StarBase database, we predicted that FHL1 was another novel downstream target of miR-105. (b) The luciferase reporter plasmids containing the wild type 3′-UTR region or mutant 3′-UTR region of FHL1 were co-transfected into A549 cells with miR-105 or in parallel with the luciferase reporter vector LINC00261-WT. (c) FHL1 expression of cancer cells was decreased in cells transfected with miR-105 mimics compared with control cells. (d) MiR-105 mimics repressed FHL1 protein expression in A549 cells. (e) MiR-105 mimics repressed FHL1 protein expression in SPCA1 cells. (f) The expression level of FHL1 in LINC00261 cells was significantly increased compared with control cells. (g) The linear correlation between the expression level of FHL1 and LINC00261 in NSCLC tissues. The results represent the average of three independent experiments. Data are presented as the mean ± standard error of the mean. *P<0.05.

MicroRNAs participate in biological process through their downstream targets. Jin *et al*. had reported that Mcl-1 was a target gene of miR-105, which contributed into NSCLC progression 11. In this work, by using StarBase Predicted data, we predicted that FHL1 was another novel downstream target of miR-105 (Fig. 4a). The luciferase assay revealed that co-transfection of FHL1-WT and miR-105 reduced luciferase activity, which could be partly restored by LINC00261-WT (Fig. 4b). RT-PCR results further demonstrated that expression of FHL1 in NSCLC cells which transfected with miR-105 mimics was significantly downregulated (Fig. 4c). Besides, it was found that FHL1 could be inhibited at protein level by miR-105 mimics through western blot assay (Fig. 4 d, e). To explore the interaction between LINC00261 and FHL1, FHL1 expression was detected in both cells and tissues. As the result, FHL1 expression of LINC00261 cells was much higher when compared to that of the control cells (Fig. 4f). The linear correlation analysis revealed that the FHL1 expression positively correlated to LINC00261 expression in NSCLC tissues (Fig. 4g).

### Overexpression of LINC00261 inhibited A549 cell growth* in vivo*

**Figure 5:**
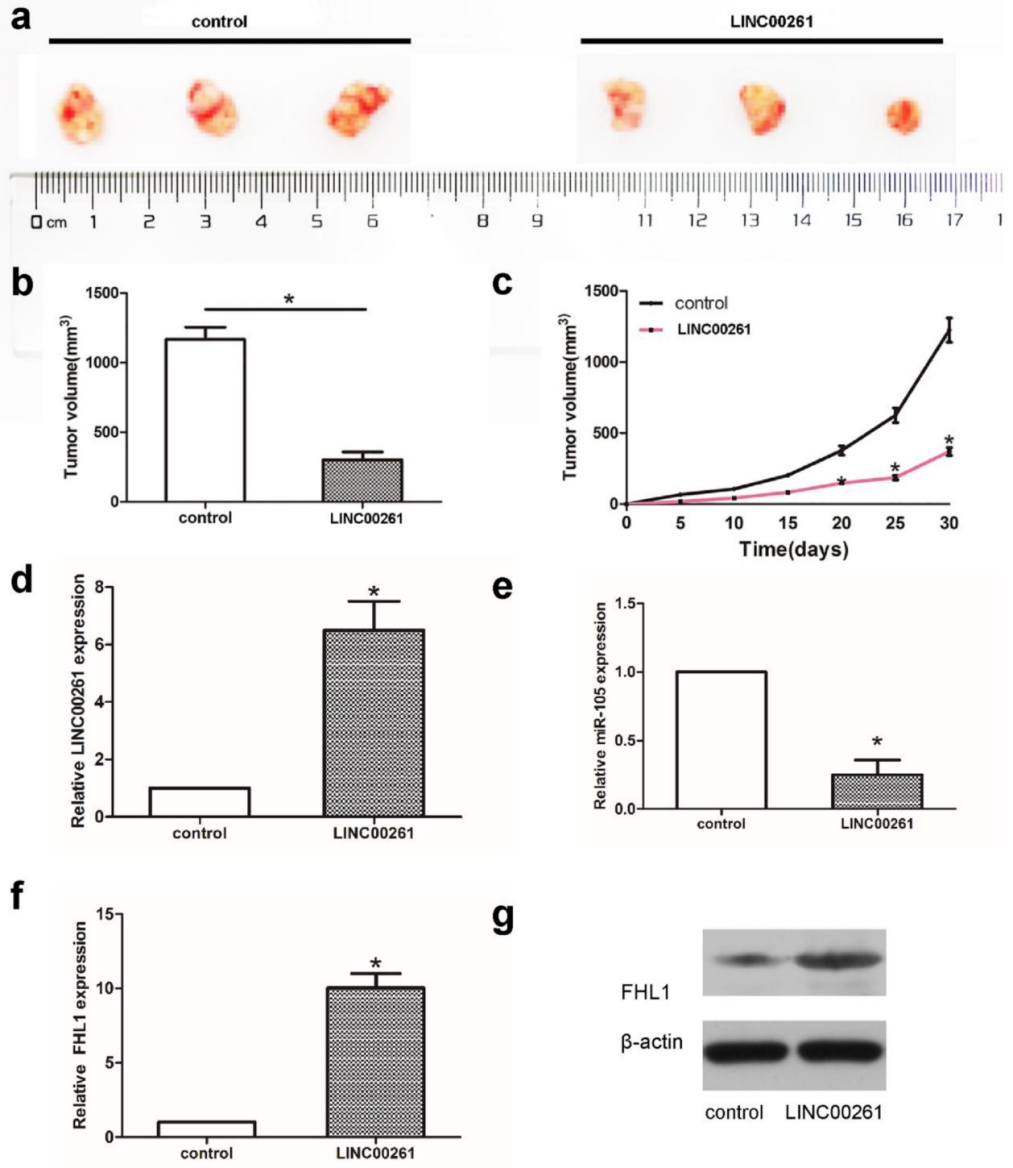
** Overexpression of LINC00261 inhibited A549 cell growth* in vivo*** (a) Tumor volume was determined by using calipers. (b, c) The tumor size in two groups from beginning to 1 month after model building. (d-f) The RNA expression levels of LINC00261, miR-105, as well as FHL1 were detected via PCR. (g) Western blot assay was adopted to detect the protein levels of FHL1 in tumor tissues from two groups. The results represent the average of three independent experiments. Data are presented as the mean ± standard error of the mean. *P<0.05.

Subcutaneous tumor mice model was established to assess cell growth. A549 cells transfected with LINC00261 or control were injected into mice subcutaneously to induce subcutaneous tumor mice model. Tumor volume was significantly smaller in LINC00261 groups compared with control groups (Fig. 5a-c). Then tumor tissues were collected to verify the expression of LINC00261 firstly. As shown in Fig. 5d, the expression of LINC00261 were overexpressed successfully in mice model of LINC00261 group. Further PCR and western blot assay showed the down-regulated of miR-105 and the higher expression of FHL1 both in RNA and protein levels in LINC00261 group (Fig. 5e-g), which revealed the effect of LINC00261 on miR-105/FHL1 axis.

## Discussion

Evidence has proved that lncRNAs participate in tumorigenesis and development. In the present study, LINC00261 was found downregulated in tissue samples and NSCLC cell lines, and the expression of LINC00261 was significant correlated with tumor size, tumor stage, lymph node metastasis and patients' prognosis. The results were consistent with previous studies, in which decreased expression of LINC00261 is a prognostic marker for NSCLC patients 12. Zhang *et al*. found that LINC00261 is a novel prognostic marker for pancreatic cancer 13. Furthermore, overexpression of LINC00261 expression could inhibit cell proliferation and invasion in NSCLC cells. Moreover, LINC00261 was also confirmed could suppress NSCLC cell growth *in vivo*. These data indicated that LINC00261 act as a tumor suppressor in NSCLC.

Recent studies reveal that LINC00261 participates in tumor progression in many cancers. In esophageal cancer, LINC00261 can induce chemosensitization to 5-fluorouracil via mediating methylation-dependent repression of DPYD 14. LINC00261 controls endometrial carcinoma progression via regulating miRNA/FOXO1 15. Moreover, LINC00261 decreases cisplatin resistance and promotes the anti-cancer effect of cisplatin in colon cancer 16. LINC00261 represses cell growth and migration in endometriosis 17. Another study revealed LINC00261 inhibits cell proliferation and invasion and promotes cell apoptosis in choriocarcinoma 18. Here, we demonstrated that LINC00261 was downregulated in NSCLC tissue samples and cells, and significantly correlated with tumor size, tumor stage, lymph node metastasis and patients' prognosis. In vitro, upregulated LINC00261 inhibited cell growth and invaded ability.

Latest studies revealed the functions of lncRNAs in NSCLC progression by binding to miRNAs. For example, LncRNA H19 acts as a ceRNA and participates in the development of NSCLC via controlling miR-107 19. LINC00978 enhances NSCLC cell proliferation and invasion via repressing miR-6754 20. It has been revealed that knockdown of lncRNA PVT1 promotes radiosensitivity in NSCLC via sponging miR-195 21. LINC00673 influences NSCLC proliferation, invasion and epithelial mesenchymal transition via sponging miR-150 22. Recent research found LINC00472 suppresses proliferation and promotes apoptosis of lung adenocarcinoma cells by modulating miR-24-3p/DEDD 23. Our study revealed that miR-105 could directly bind to LINC00261 through a luciferase assay. And miR-105 was significantly enriched by LINC00261 RIP assay. In addition, miR-105 expression could be downregulated with overexpression of LINC00261. In NSCLC tissues, the expression of miR-105 was downregulated by LINC00261 expression. All the results above suggest that LINC00261 might inhibit tumorigenesis of NSCLC via sponging miR-105.

High miR-105 expression was associated with aggressive phenotype of colorectal cancer, and the enhanced expression of miR-105 was required for TNF-α-induced epithelial-mesenchymal transition (EMT) 24. Cancer-secreted miR-105 destroyed vascular endothelial barriers to promote metastasis 25. MiR-105 promoted EMT of NSCLC via upregulating Mcl-1 26. However, miR-105 suppressed cell proliferation and inhibited PI3K/AKT signaling in hepatocellular carcinoma 27. miR-105 inhibited prostate tumor growth by suppressing CDK6 levels 28. Reduced miR-105-1 was related to poor survival of patients with NSCLC 29. Collectively, this inconsistent finding may be due to the tumor type or contamination of normal tissue in the tumor samples or lack of samples or others. Thus, additional studies are required to clarify the role of miR-105 in NSCLC. MicroRNAs participate in biological process through their downstream targets. Jin et al. had reported that Mcl-1 was a target gene of miR-105, which contributed into NSCLC progression 11. In this work, we identified that FHL1 was another novel downstream target of miR-105. The four and a half LIM domains (FHL) family participates in cell proliferation, apoptosis and differentiation in skeletal and cardiac muscle growth, and the member FHL1, located on chromosome Xq26 30, 31. Notably, FHL1 as a tumor suppressor gene is reduced in the malignant cancers, including gastric 32, 33, breast, kidney and prostate cancers 34. Niu et al. confirmed that FHL1 was downregulation and has an inhibitory role in the progression of lung cancer 35. miRNAs can regulate biological behaviors by targeting one or more genes, which indicates the complicated processes of tumors are owing to not only one molecular but also two or more factors, even a network. Thus, further understanding the regulatory mechanism of multiple molecules in tumors progression is needed.

## Conclusion

Above data identified that in NSCLC tissues, LINC00261 was remarkably downregulated and was better related to disease-free survival of NSCLC patients. Besides, LINC00261 could inhibit cell proliferation and invasion through miR-105/FHL1 axis both confirmed* in vitro* and *in vivo*. These findings suggested that LINC00261 might contribute to therapy for NSCLC as a candidate target.

## Figures and Tables

**Figure 3:**
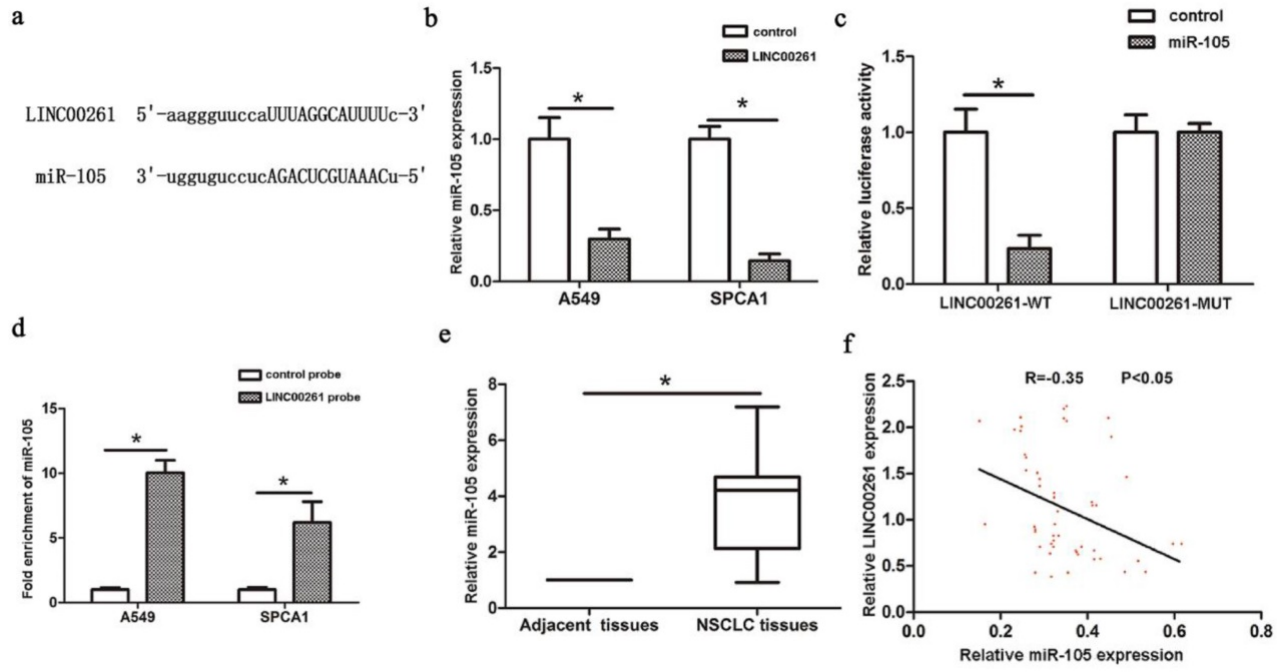
** Interaction between LINC00261 and miR-105** (a) StarBase Predicted data was used to find the miRNAs that contained complementary base with LINC00261. (b) MiR-105 expression was decreased in LINC00261 group compared with control group. (c) Co-transfection of miR-105 and LINC00261-WT in A549 cells strongly decreased the luciferase activity, while co-transfection of miR-105 and LINC00261-MUT did not change the luciferase activity either. (d) MiR-105 was significantly enriched by RNA immunoprecipitation (RIP) assay in the LINC00261 group compared with control. (e) MiR-105 was significantly upregulated in NSCLC tissues compared with adjacent tissues. (f) The linear correlation between the expression levels of miR-105 and LINC00261 in NSCLC tissues. The results represent the average of three independent experiments Data are presented as the mean ± standard error of the mean. *P<0.05.

**Table 1:** Correlation between LINC00261 expression and clinicopathological characteristics in NSCLC patients

Characteristics	Patients	Expression of LINC00261		*P*-value
		High- LINC00261	Low- LINC00261	
Total	60	27	33	
**Age (years)**				0.693
≤50	25	12	13	
>50	35	15	20	
**Gender**				0.100
Male	27	9	18	
Female	33	18	15	
**TNM stage**				0.017*
I-II	32	19	13	
III-IV	28	8	20	
**Tumor size**				0.020*
<3cm	30	18	12	
>3cm	30	9	21	
**Lymphatic metastasis**				0.024*
No	26	16	10	
Yes	34	11	23	
